# Chapter IV: Rheumatism

**Published:** 1883

**Authors:** 


### CHAPTER IV.

### RHEUMATISM.

By this term we indicate an extended class of affections by no means easily brought within the brief and explicit terms of scientific definition. The difficulty is not so much in the vagueness of the subject as in its extent and the varied character of cases brought within the domain ascribed to it. When we suffer the pains of rheumatism we find them neither shadowy nor uncertain, but when we attempt to define what we mean by the word, to present the elements belonging to the genus, found in all its examples and found nowhere else, we fail, even after much labor, to produce anything satisfactory, and may be compelled to sit down, as the writer now is, to the employment of the word, trusting to its general use by readers for a recognition of the fact that we refer to that numerous class of painful affections which are gathered under this denomination, for the good reason that it has not been so convenient to place them under any other. When he says *Rheumatism*, the reader thinks he means a definite thing enough, and if perchance he has felt its grip he is quite sure of it. Will he be so good as to tell us what he means, and in terms which shall equally describe the tortures of his ten dear neighbors and friends who also had suffered, and who certainly came to sympathize with him, and each to bring the knowledge of a "certain cure." If he will do this, he will have made an addition to the literature of this subject which will deserve to be remembered. If this definition were essential to that understanding of cases as they occur in practice, requisite to their intelligent treatment and speedy cure, it would be a matter for the deepest regret. But as the relationship of curatives to diseases has not been made to depend on strictly scientific definitions, however valuable these may be, and as the chief duty of the physician, in these, as in all other cases, is to find the curative, and as definition, if given, cannot materially aid him in the discharge of this duty, its consideration may for the present be safely waived. The improvements in nomenclature may hereafter make definitions more possible, the signification of terms used more explicit, and thereby enhance convenience in the interchange of ideas, but whether increased certainty of cure, or an abridged duration of the disease, would necessarily result may well be doubted.

In order to a certain and speedy cure of rheumatism, it is essential to begin with that first of all requisites to intelligent and successful treatment of diseases, a strict individualization of the case. It will be a help toward this, if at the outset the case can be referred to either of the following classes: With fever; without fever; swelling with redness and heat; without swelling; swelling with œdema; pains aggravated by motion; pains relieved by motion; pains suddenly shifting from place to place; pains aggravated by change of weather, especially on the approach of a storm. The exciting cause, if known, should be carefully considered, as this is often an important element in the individualization, and also the *quality* of the pain. The time and circumstances of aggravation are also matter for the strictest inquiry. The character of the tissue invaded—is it muscular, tendonous, ligamentous, membranous, or a mixture of these?

